# Insulin-like growth factor I promotes cord blood T cell maturation through monocytes and inhibits their apoptosis in part through interleukin-6

**DOI:** 10.1186/1471-2172-9-74

**Published:** 2008-12-17

**Authors:** Helen KW Law, Wenwei Tu, Enmei Liu, Yu Lung Lau

**Affiliations:** 1 Department of Paediatrics and Adolescent Medicine, Li Ka Shing Faculty of Medicine, The University of Hong Kong, Queen Mary Hospital, Pokfulam, Hong Kong, PR China; 2 Division of Respiratory Disorders, Children's Hospital, Chongqing University of Medical Sciences, Chongqing, PR China; 3 Department of Paediatrics and Adolescent Medicine, The University of Hong Kong, New Clinical Building, Queen Mary Hospital, Pokfulam, Hong Kong, PR China

## Abstract

**Background:**

The functional immaturity of T cells contributes to the susceptibility of neonates to infections and the less severe graft-versus-host disease associated with cord blood (CB) transplantation. We have previously reported that insulin-like growth factor – I (IGF-I) promotes the phytohaemagglutinin (PHA)-induced CB T cell maturation and inhibits their apoptosis in mononuclear cell (MC) culture. We hypothesized that the effects of IGF-I may be mediated by accessory cells and soluble factors.

**Results:**

This study showed that the kinetics of PHA-induced maturation in purified CD3+ T cell was delayed compared to that in CBMC. The addition of autologous CD14+ monocytes increased T cell maturation and potentiated the effect of IGF-I. The addition of IL-6 had no effect on CB T cell maturation but it reduced PHA-induced apoptosis significantly. We further demonstrated that the neutralisation of IL-6 in CBMC culture partially abrogated the anti-apoptotic effect of IGF-1 on T cells. The anti-apoptotic effect of IL-6 was not mediated via the reduction of Fas expression in T cell subsets.

**Conclusion:**

Our results suggested that the maturation effect of IGF-1 is partially mediated by monocytes and the anti-apoptotic effect in part via IL-6. Further investigation is needed to explore the therapeutic use of IGF-I in enhancing neonatal immunity.

## Background

Neonates, in particular pre-term babies, have increased susceptibility to infections. It has been hypothesised that the functional immaturity of neonates may be the results of a number of factors, including the immaturity of the cellular component, the lack of previous exposure to antigens, and the uniqueness of the cytokine environment [1,2]. There is increasing evidence that the survival of lymphocytes during sepsis may improve the disease outcome of the host [3]. Therefore, cytokines with the potential to promote the activation of T cells and their survival may have great clinical application.

Insulin-like growth factor – I (IGF-I), which is known to be the mediator for many growth promoting effects of growth hormone, has also shown to have a direct influence on the maintenance of normal immune function [4,5]. It is a lymphopoietic cytokine which has profound stimulatory effects on lymphopoiesis *in vitro *[6-8] and *in vivo *[4]. Our previous study on cord blood mononuclear cell (CBMC) culture demonstrated that IGF-I has important immunobiological effects on proliferation, IL-6 and IFN-γ production [9] and increase in telomerase activity [10]. IGF-1 also promotes maturation and inhibits the spontaneous and PHA-induced apoptosis of CB T cells [11]. It reduces the expression of Fas and protects T cells from Fas ligand induced apoptosis in CBMC culture [11]. These previous studies were based on a mixed population of cells, in which the direct effect of IGF-1 on T cell and the roles of non-T cells have not been investigated. The objective of the current study is to delineate the effect of IGF-I in purified CD3+ T cell culture and to determine the roles of accessory cells and soluble factors in mediating the effects of IGF-I.

CD45, the leukocyte-common antigen, is a family of transmembrane glycoprotein with tyrosine phosphatase activity. Various isoforms are formed by the alternative splicing of three consecutive NH2-terminal exons of the CD45 transcript and are expressed differentially on haemopoietic cells. The expression of different isoforms is highly regulated but the detailed mechanisms are still unknown[12]. CD45RA and CD45RO are two well-studied isoforms detected on naïve/resting and memory/activated T lymphocytes respectively. Neonates are immunologically naive with little exposure to microorganisms and foreign antigens. Therefore, most neonatal T cells are naive and express CD45RA, whereas both naive CD45RA+ and mature CD45RO+ T cells are of approximately equal numbers in adult peripheral blood mononuclear cells [13,14]. Upon the exposure of appropriate *in vitro *stimulation, CD45RA+ cells can acquire phenotypic and functional characteristics of CD45RO+ cells, in particular the helper cell function and cytokine production [15-17]. The expression of CD45RA and CD45RO are, however, not mutually exclusive [18,19] and interconversion of the CD45 isoform *in vivo *has been reported [20]. It has been suggested that the dual positive CD45RA+CD45RO+ cells represent a population of transitional cells which contain primed T cells, which in the absence of antigenic stimulation may re-aquire a naïve phenotype [21].

It has been recognised previously that accessory cells, in particular monocytes, play a crucial role in the regulation of T lymphocyte differentiation and activation [22-24]. Monocytes express antigen presenting and co-stimulatory molecules (such as MHC Class II molecules, CD1, CD40, CD80 and CD86) and release cytokines (such as TNF-α, IL-1 and IL-6) that act on T lymphocytes. We therefore extended our experiments to study the role of monocytes and IL-6 in the maturation of CB T cells in order to determine the underlying mechanisms mediating the effect of IGF-I.

IL-6 has been reported to be an anti-apoptotic cytokine which can prevent T cells apoptosis. However, the reports are restricted to murine studies [25,26]. Since IGF-I have also been reported to stimulate the release of cytokines from monocytes [27] and CBMC [9], we further investigated if IL-6 has a direct anti-apoptotic effect on purified CD3+ T cells. To confirm the role played by IL-6 in our previous reports, we neutralised IL-6 in CBMC culture by monoclonal antibodies. The possible anti-apoptotic mechanism involving the expression of Fas on different subsets of T cells was also investigated. This is the first report which provide evidences that monocytes play a role in mediating the maturation effect of IGF-I, whereas the anti-apoptotic effect of IGF-I may be mediated through the release of IL-6 from accessory cells.

## Methods

### Cell Preparation

Human umbilical cord blood samples were collected from the placentae of normal full-term uncomplicated pregnancy. Informed consent was obtained from the mothers prior to delivery based on the guidelines approved by the Institutional Review Board of the University of Hong Kong/Hospital Authority Hong Kong West Cluster. Ficoll Hypaque density separation (Pharmacia Fine Chemicals, NJ, USA) was performed to isolate the low-density mononuclear cells.

CD3+ and CD14+ cells were isolated by positive immunomagnetic separation according to the manufacturer's recommendation with slight modifications (Miltenyi Biotec GmbH, Bergisch-Gladbach, Germany). High purity of CD3+ (95.28 ± 0.71%; n = 27) and CD14+ (84.21 ± 1.64%; n = 8) cells were obtained consistently.

### Cell Culture

All culture was performed in a serum free, insulin free medium (DMEM-F12, Sigma Chemical Co. St Louis, MO, USA) supplemented with 50 i.u./mL penicillin and 50 μg/mL streptomycin (Gibco BRL, Life Technologies Inc., NY, USA). IGF-I (100 ng/mL, R&D System, Minneapolis, USA), PHA (1 μg/mL, Sigma Chemical Co. St Louis, MO, USA), and IL-6 (1–50 ng/mL, R&D System, Minneapolis, USA) were added in various combinations as described in the results. The concentrations of IGF-I and PHA used have been titrated in previous studies.

CBMC and purified CB CD3+ T cells were cultured at concentrations of 1 × 10^6 ^and 4 × 10^5 ^cells/mL respectively. In some experiments autologous CB CD14+ monocytes were added to CD3+ T cells at fixed ratios. The cultures were incubated at 37°C in 5% CO_2 _with 100% humidified air and harvested on Day 3, 6 and 9.

To neutralise the effect of IL-6, CBMC were cultured for 4 days at 1 × 10^6 ^cells/mL in DMEM-F12 containing IGF-1 (100 ng/mL) + PHA (1 μg/mL) and 1–20 μg/mL of anti-IL-6 neutralising antibodies (R&D System, Minneapolis, USA).

### Flow cytometric analysis

The phenotype of cells was analysed by 3-colour flow cytometry. Briefly, cells were collected at defined time points and stained with different combinations of FITC, PE or PC5 conjugated monoclonal antibodies for isotype control, CD3, CD4, CD8, CD45RA (ALB11), CD45RO (UCHL1), and CD95 (Beckman Coulter, Immunotech, Marseille, Cedex, France), and analysed within 24 hours. Apoptosis in different T cell subsets was detected by Annexin-V apoptosis kits (Beckman Coulter, Immunotech, Marseille, Cedex, France) within 10 minutes of staining. Using the Beckman Coulter Elite flow cytometer (Beckman Coulter Electronics, FL, USA), ten thousand events per sample were collected into listmode files and analysed by the Prudue University WinMDI software. The analysis of T lymphocytes in the co-culture system was achieved by careful setting of flow cytometer parameters and consistent gating based on the scatter characteristics of lymphocytes. In this region, over 90% of cells are CD3+ and no CD14+ cells were detected.

### Cytokine assay

Cell culture supernatant was collected at defined time points and the concentration of IL-6 determined by enzyme-linked immunosorbent assay (ELISA, Quantikit, R&D System, Minneapolis, USA). The detection limit was 15.6 pg/mL.

### Statistical Analysis

All percentages are presented as mean ± standard error of independent cases unless otherwise stated. The non-parametric Wilcoxon signed rank sum test was used to determine the difference between paired groups; whereas, the non-parametric Mann-Whitney U statistic test was used for unpaired groups. The conventional significance level (α) of 0.05 was used.

## Results

### IGF-I significantly promoted PHA-induced CD45 isoform conversion for CD4+ and CD8+ subsets in CBMC culture

We have previously reported the effect of IGF-I on CD45 isoform conversion in CD3+ cells in CBMC culture [11]. In this study, we further investigated the effect of IGF-I on CD4+ and CD8+ subsets. In CBMC culture, the majority of T cells in CBMC are naïve cells (CD4+CD45RA+CD45RO-: 78.1 ± 1.0%; CD8+CD45RA+CD45RO-: 89.1 ± 1.9%; n = 8). A small proportion of cells expressed the intermediate phenotype (CD4+CD45RA+CD45RO+: 2.8 ± 0.4%; CD8+CD45RA+CD45RO+: 2.4 ± 0.7%; n = 8) or the mature phenotype (CD4+CD45RA-CD45RO+: 10.6 ± 1.4%; CD8+CD45RA-CD45RO+: 3.4 ± 1.0%; n = 8).

**Figure 1:**
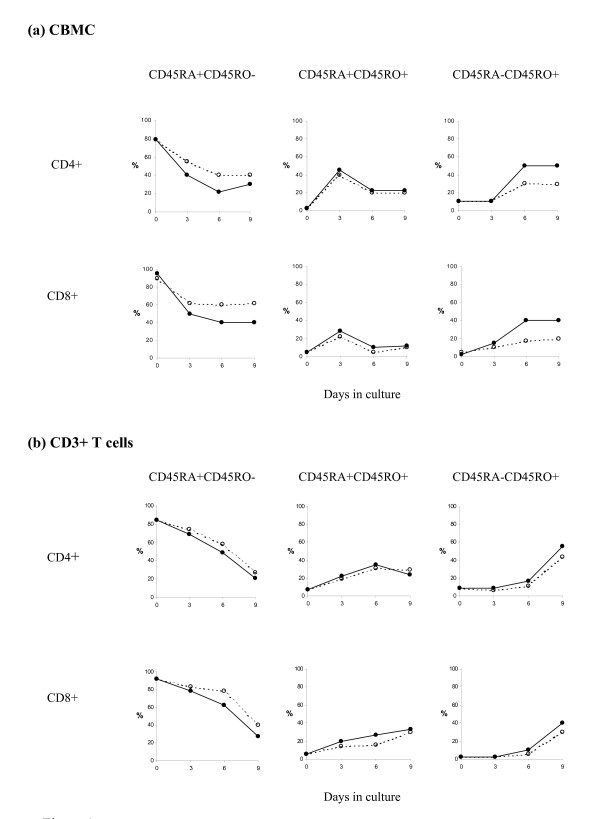
**The kinetics of CD45 isoform changes for CD4+ T helper cells and CD8+ cytotoxic T cells in (a) CBMC culture; and, (b) purified CD3+ T cell culture.** CBMC (a) or CD3+ T cells (b) were incubated for different period with PHA (1 μg/ml ----○----) alone or PHA + IGF-I (100 ng/ml —●—). CD45 isoform expression was determined by three-colour flow cytometry. The kinetics of transition from naïve to mature/memory phenotype in CD4+ and CD8+ cells were similar. In CBMC, IGF-I promoted the PHA-induced maturation of CD45 isoforms. In CD3+ T cells, the maturation process was delayed and IGF-I only slightly promoted the PHA-induced maturation of CD45 isoforms. Results shown are representatives of 5 similar independent cases in each group.

When stimulated by PHA alone, the CD4+CD45RA+CD45RO- cells in CBMC culture decreased gradually from about 80% to 40% from Day 0 to Day 9 (Fig 1a, upper panel, dotted line). There was a complementary increase in the CD4+CD45RA+CD45RO+ dual positive population which reached a maximum level of about 40% on Day 3 and declined to about 20% on Day 6. The proportion of mature memory cells (CD4+CD45RA-CD45RO+) remained at about 10% for the first 3 days of activation before increasing to about 30% by Day 6, and then maintained at that level until Day 9.

Similar to the T helper cells, the percentage of CD8+CD45RA+CD45RO- cytotoxic T cells decreased sharply after Day 3 of PHA activation, then maintained at about 60% through to Day 9 (Fig 1a, lower panel, dotted line). The CD8+CD45RA+CD45RO+ double-positive cells was highest on Day 3 (~30%), and then maintained at rather low level of ~10% from Day 6 to Day 9. The CD45RO single-positive CD8+ cells (CD8+CD45RA-CD45RO+) increased gradually over the 9 days of culture.

**Figure 2:**
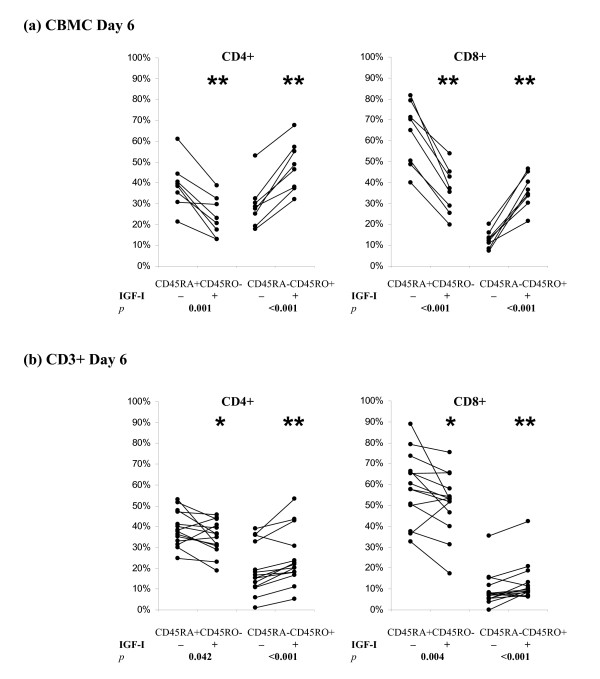
**Effects of IGF-I on PHA-induced changes in CD45 isoform expression on CD4+ T helper cells and CD8+ cytotoxic T cells in (a) CBMC culture; and, (b) purified CD3+ T cell culture**. CBMC (a) or CD3+ T cells (b) were incubated with PHA (1 μg/ml) ± IGF-I (100 ng/ml) for 6 days. IGF-I significantly promoted the PHA-induced down-regulation of naïve T cells (CD45RA+CD45RO-) and up-regulation of mature T cells (CD45RA-CD45RO+). Results shown are from 8 and 14 independent cases of CBMC and CD3+ T cells respectively.

The addition of IGF-I to the CBMC did not change the general kinetic of CD45RA to CD45RO conversion but significantly promoted the decline in the proportion of naïve T cells and the increase in memory/mature phenotype (Fig 1a, solid lines). The effects of IGF-I on CD4+ and CD8+ cells in CBMC cultures were similar. To consolidate our observation, we compared the percentages of naïve and mature T helper and cytotoxic T cells in Day 6 culture with PHA ± IGF-I (Fig 2a). IGF-I significantly decreased the percentages of CD45RA+ cells (p < 0.001; n = 8) and increased the percentages of CD45RO+ cells (p < 0.001; n = 8).

### The kinetics of PHA-induced CD45 isoform conversion in purified CD3+ T cell culture was different from that in CBMC culture

Immunomagnetic separation yield highly purified CD3+ population in which a high majority of the CB T cells are naïve, bearing the CD45RA+ phenotype (CD4+CD45RA+CD45RO-: 89.8 ± 2.5%; CD8+CD45RA+CD45RO-: 94.8 ± 1.6%; n = 8). A very small proportion of the cells co-expressed both CD45 isoforms (CD4+CD45RA+CD45RO+: 2.5 ± 3.6%; CD8+CD45RA+CD45RO+: 2.3 ± 1.1%; n = 8) and only a minority of cells expressed the CD45RO+ memory/mature phenotype (CD4+CD45RA-CD45RO+: 7.7 ± 3.8%; CD8+D45RA-CD45RO+: 3.0 ± 0.9%; n = 8).

When the purified CD3+ T cells were cultured in serum free medium or IGF-I alone, there was no spontaneous conversion of CD45 isoforms and too few cells survive after day 6 for phenotypic analysis (data not shown). Similar to the observation in CBMC, PHA alone induced the conversion of CD45RA+ cells into CD45RO+ cells in the CD3+ cell culture (Fig 1b, dotted lines). However, there was a delay in the conversion of naïve cells to the dual positive cells and consequently the delay in the maturation of cells into the CD45RO+ phenotype. The percentage of CD45RA+CD45RO+ cells reached maximum on or after Day 6 and the percentage of CD45RA-CD45RO+ cells was maximum on Day 9 in CD3+ cell culture.

The kinetics of CD45 isoform switching in culture with PHA+IGF-I was very similar to that observed in CD3+ T cells cultured with PHA alone (Fig 1b). IGF-I only promoted a slight increase (<10%) in T cell maturation (Fig 1b, solid lines). Summarising data from 14 experiments (Figure 2b), we confirmed that IGF-I significantly promoted PHA-induced T cell maturation in CD3+ T cell culture (Fig 2b). However, the effect of IGF-I in purified CD3+ T cell culture was weaker than that observed in CBMC culture suggesting that factors present in the CBMC culture but absent in CD3+ T cells culture may be critical mediators of the effect of IGF-I.

### Monocytes potentiated the effect of IGF-I on PHA-induced CD45 isoform conversion

**Figure 3:**
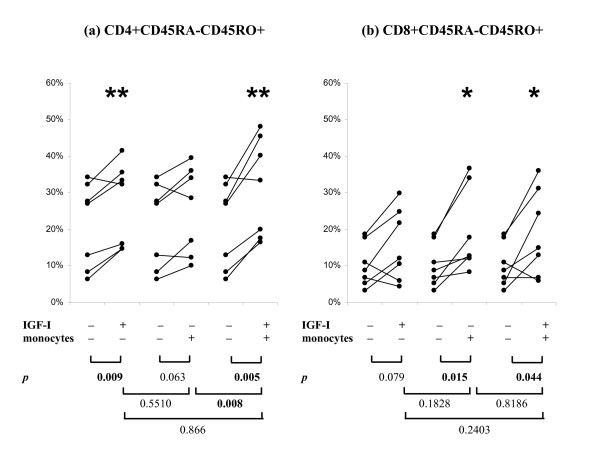
**Effects of monocytes in potentiating the effects of IGF-I on PHA-induced changes in CD45 isoform expression in purified CD3+ T cell culture**. CD3+ T cells were incubated with PHA (1 μg/ml) alone, PHA + IGF-I (100 ng/ml), PHA + autologous CD14+ monocytes (monocytes: T cells ratio = 1:4), or PHA + IGF-I + monocytes for 6 days. The addition of monocytes promoted CD4+ and CD8+ T cells maturation and the effect was further increased in the presence of IGF-I. Results shown are from 7 independent cases of CD3+ T cells respectively.

To determine if monocytes play a role in the conversion of CD45 isoform, we added autologous CD14+ monocytes back to the purified CD3+ T cell cultures at monocytes to T cells ratios of 1:4 based on our analysis of the mean CD14+:CD3+ ratio in CBMC (1:3.8 ± 2.5; n = 26). The effects of monocytes and IGF-I on PHA-induced CD45 isoform conversion is summarised in Figure 3. Similar to previous experiments, the addition of IGF-I significantly promoted CD4+ cell maturation (Figure 3a, 21.3% vs 26.9% in PHA vs PHA+IGF-I respectively; p = 0.009; n = 7). The addition of monocytes alone increased the percentage of mature cells but did not reach statistical significance (21.3% vs 25.3% in PHA vs PHA+monocyte respectively; p = 0.063). In the presence of both IGF-I and monocytes, the CD4+CD45RA-CD45RO+ cells reached 31.6%. This is significantly higher than PHA alone (p = 0.005) or PHA+monocyte (p = 0.008) but not PHA+IGF-I (p = 0.0866). There was a trend of increased maturation and it is due to sample variation that the difference did not reach statistical significance.

In CD8+ cells (Figure 3b), the addition of IGF-I promoted maturation but the increase did not reach statistical significance (15.6% vs 10.3% in PHA vs PHA+IGF-I respectively; p = 0.079; n = 7). The addition of monocytes alone significantly increased the percentage of mature cells (15.6% vs 19.2% in PHA vs PHA+monocyte respectively; p = 0.015). In the presence of both IGF-I and monocytes, the CD4+CD45RA-CD45RO+ cells reached 18.9%. This is significantly higher than PHA alone (p = 0.044) but not significantly different from PHA+IGF-I (p = 0.2403) and PHA+monocytes (p = 0.8186).

### IL-6 did not modulate PHA-induced CD45 isoform conversion in CD3+ T cell culture

**Figure 4:**
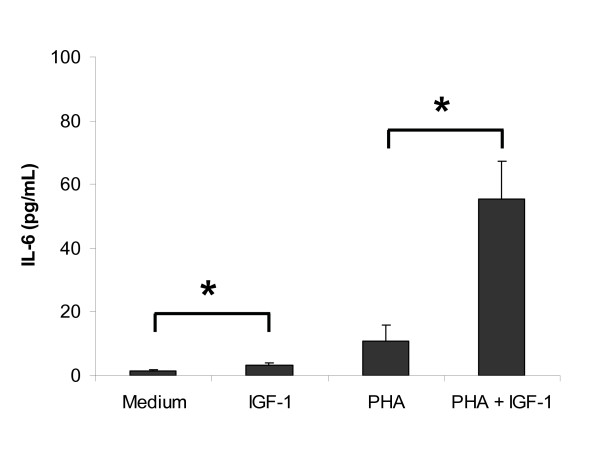
**Effect of IGF-1 on IL-6 production in CB CD3+ T cell culture**. The level of IL-6 detected in the purified T cell culture was much lower than that observed previously in CBMC. IGF-1 alone induced a slight but significant increase in IL-6 (n = 7; * p = 0.04). PHA alone did not induce IL-6 production (p = 0.44). IGF-1 induced a significant increase in PHA-induced IL-6 production. Results shown are mean ± SEM of 7 independent cases (* p = 0.02).

In our previous report, we have demonstrated that IGF-I increases the protein production and mRNA expression of IL-6 in PHA-stimulated CBMC culture [9]. In this study, we analysed the concentrations of IL-6 in cell supernatant collected from Day 3 CB CD3+ T cell cultures (Figure 4). The level of IL-6 (range 1 – 120 pg/mL, n = 7) was much lower than that observed previously in CBMC (range: 600 pg/mL – 4000 pg/mL). IGF-1 alone stimulated a slight but significant increase in IL-6 (medium = 1.47 ± 0.26 pg/mL vs IGF-1 = 3.27 ± 0.64 pg/mL, p = 0.04). PHA alone also stimulated IL-6 production but due to sample variation, the changes did not reach statistical significance (PHA = 10.76 ± 5.02 pg/mL, p = 0.22). IGF-1 induced a significant increase in PHA-induced IL-6 production (PHA+IGF-1 = 55.39 ± 11.71 pg/mL; p = 0.02). The addition of higher concentration of PHA (10 μg/mL) did not increase the production of IL-6 in T cell culture (data not shown). We determined the role of IL-6 in T cell maturation by adding titrated dose of IL-6 (1–50 ng/mL) to PHA-stimulated CD3+ T cells culture. The addition of IL-6 within this range did not affect the maturation of T helper cells/cytotoxic T cells in PHA-stimulated CD3+ T cell culture (data not shown, n = 3, p > 0.05).

### IL-6 was more efficient than IGF-I in inhibiting PHA-induced apoptosis in CD3+ T cell culture

We further investigated the effects of IGF-I and IL-6 on apoptosis in the CD4+ and CD8+ subsets in the purified CD3+ cell culture. In cultures with medium or IGF-1 alone, too few cells survived on Day 4 for apoptosis analysis (data not shown). Similar to the observation in CBMC, PHA alone induced mainly early apoptosis of the T cell culture (AV+PI- % = 70.8 ± 3.8%; n = 9). Only a minor proportion of cells were in late apoptosis (AV+PI+ % = 5.2 ± 1.0%; n = 9). IGF-1 induced similar level of apoptosis in the whole population (AV+PI- % = 69.1 ± 5.1%; p = 0.57; AV+PI+ % = 3.8 ± 0.5%; p = 0.31; n = 9).

**Figure 5:**
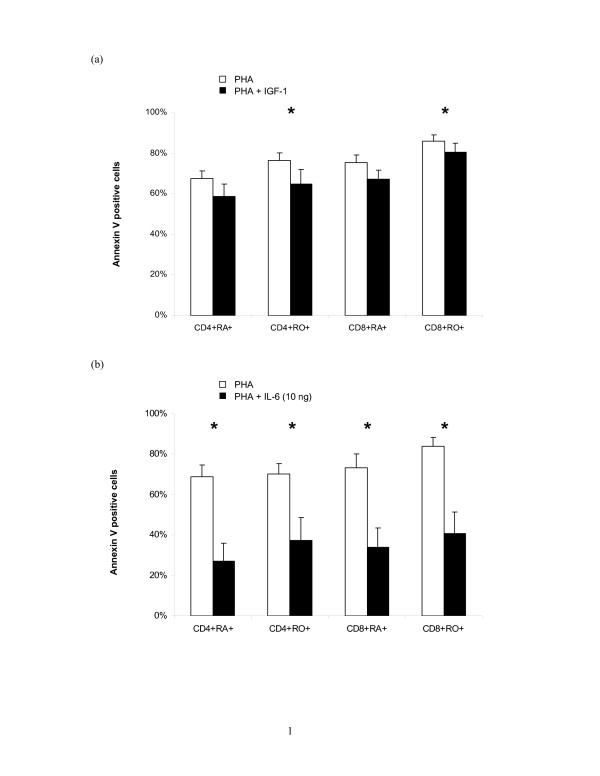
**Effects of IGF-1 and IL-6 on PHA-induced apoptosis in purified CB CD3+ T cell culture**. CB CD3+ T cells were cultured with (a) PHA (1 μg/mL) ± IGF-1 (100 ng/mL) or (b) PHA (1 μg/mL) ± IL-6 (10 ng/mL) for 4 days. Both IGF-1 and IL-6 significantly inhibited PHA-induced apoptosis in T cell subsets. Results shown are mean ± SEM of 9 and 4 independent cases of CD3+ T cells respectively (* p < 0.05).

Since majority of the cells are PI-, we omitted the PI staining and determined the percentage of AV+ cells in each T cell subset as the index of apoptosis using 3 colour flow cytometry (Figure 5a). The addition of IGF-1 caused a slight reduction of apoptosis the reduction only reached statistical significance in the CD4+CD45RO+ and CD8+CD45RO+ subsets (both p = 0.002; n = 9).

Since the observed anti-apoptotic effect of IGF-1 in the purified CD3+ T cells culture was much lower than that previously observed in mixed population of CBMC, we studied the effect of IL-6 on PHA-induced apoptosis in CD3+ T cells. In our preliminary experiments, IL-6 (1–50 ng/mL) significantly inhibited PHA-induced apoptosis in a dose dependant manner reaching maximum reduction at 10 ng/mL (data not shown). IL-6 (10 ng/mL) significantly lowered the percentage of early apoptotic cells in PHA-induced T cells from 63.8 ± 5.4% to 18.5 ± 2.2% (p = 0.029; n = 4). There was very low percentages of late apoptotic cells (PHA = 2.7 ± 0.8% and PHA+IL-6 = 1.6 ± 0.3%). As shown in Figure 5b, the percentages of AV+ cells in all the T cell subsets were reduced by about 40% in all T cell subsets and there was no preferential protection for naïve versus memory T helper cells nor cytotoxic T cells.

### Neutralisation of IL-6 in CBMC culture abrogates the anti-apoptotic effect of IGF-1

**Figure 6:**
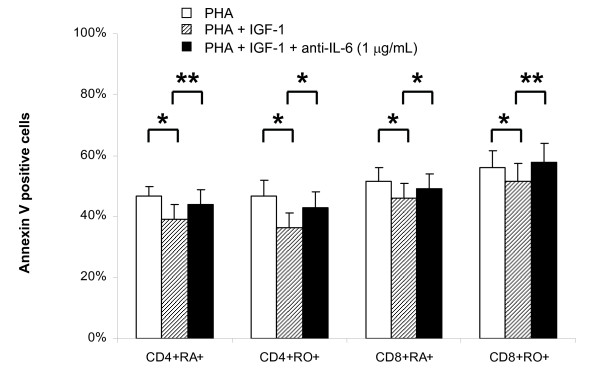
**Effect of neutralising anti-IL-6 antibodies on IGF-1 stimulated PHA-induced apoptosis in CBMC culture**. Neutralising anti-IL-6 antibodies (1 μg/mL) were added to CBMC cultured in serum free medium with PHA (1 μg/mL) + IGF-1 (100 ng/mL). Anti-IL-6 antibodies abrogated the anti-apoptotic effects of IGF-1. Results shown are mean ± SEM of 7 independent cases (* p < 0.05).

To confirm the role played by IL-6, we neutralised IL-6 in mixed CBMC culture by anti-IL-6 antibodies (1–20 μg/mL) and determined the proportion of apoptotic cells by flow cytometry. Neutralisation of IL-6 by 1–20 μg/mL of anti IL-6 antibodies resulted in similar increase in the percentages of apoptotic cells in CBMC culture (data not shown). As low as 1 μg/mL of anti-IL-6 can significantly increase the percentage of early apoptotic cells (PHA+IGF-1 = 48.6 ± 5.8% and PHA+IGF-1+anti-IL-6 = 53.7 ± 6.5%; p = 0.0156; n = 7) but not the late apoptotic cells (PHA+IGF-1 = 1.5 ± 0.2% and PHA+IGF-1+anti-IL-6 = 1.4 ± 0.2%; p = 0.3125; n = 7). The changes was particularly significant in the CD4+CD45RA+ and CD8+CD45RO+ T cells subsets (both p < 0.001; n = 7; Figure 6).

### The anti-apoptotic effect of IL-6 on T cell subsets in CD3+ culture was not mediated by reduction in Fas expression

**Figure 7:**
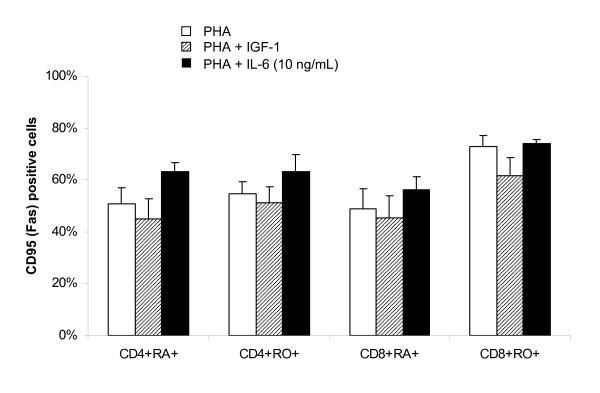
**Effect of IGF-1 and IL-6 on PHA-induced CD95 (Fas) expression on T cell subsets in CB CD3+ T cell culture**. CB CD3+ T cells were cultured with PHA (1 μg/mL) ± IGF-1 (100 ng/mL) or IL-6 (10 ng/mL) for 4 days and the expression of CD95 (Fas) on T cell subsets determined by 3 colour flow cytometry. IGF-1 slightly downregulated CD95 expression on T cell subsets while IL-6 slightly upregulated CD95 expression on T cell subsets. Results shown are mean ± SEM of 5 independent cases (* p < 0.05).

Increasing number of pathways involving various molecules have been reported for programmed cell death [28]. We have previously reported that IGF-1 down-regulates the expression of Fas on T cells in CBMC culture and protects cells from Fas ligand induced apoptosis [11]. In this study, we also analysed the effects of IGF-1 and IL-6 on the expression of Fas on PHA stimulated CD3+ T cells. IGF-1 slightly reduced the expression of Fas on PHA stimulated T cells (Figure 7) but the reduction did not reach statistical significance (p = 0.55, 0.55, 0.84, and 0.55 for CD4+CD45RA+, CD4+CD45RO+, CD8+CD45RA+, and CD8+CD45RO+ respectively; n = 5). The addition of IL-6 induced a slight but statistical insignificant increase in Fas expression in all T cell subsets (p = 0.15, 0.31, 0.31, and 0.69 for CD4+CD45RA+, CD4+CD45RO+, CD8+CD45RA+, and CD8+CD45RO+ respectively; n = 5).

## Discussion

The functional immaturity of T cells in neonates has been considered as one of the major contributing factors for the susceptibility of neonates to infections (Kovarik et al, 1998) as well as less severe graft-versus-host disease (GvHD) associated with cord blood stem cell transplant [29]. Probably as a result of antigen exposure, there is an age-related decrease in "virgin, non-antigen primed, naïve CD45RA+ T cells" and increase in "mature, activated, memory CD45RO+ T cells" [30-33]. The expression of CD45 isoforms is also regulated by mitogenic and antigenic activation. When stimulated with mitogens such as PHA or Con A *in vitro*, CD45RA+ T cells lose CD45RA expression and gain CD45RO expression [34-36]. Cytokines that can promote the maturation of T cells would have a high potential for clinical application in the management of infection in neonates.

IGF-I has been reported to have profound positive effects on immune function [7] and most recently IGF-I has shown to increase survival in a murine sepsis model [37]. We have previously reported the positive effects of IGF-I on PHA-stimulated CBMC in terms of cytokine production [9], proliferation [10], and survival [11]. In this study, we further demonstrated that IGF-I can significantly promote the PHA-induced cord blood T helper cell and cytotoxic T cell maturation in CBMC but less efficiently in the purified CD3+ T cell culture in which accessory cells are absent (Figure 1).

Similar to a previous report on CD45 isoform conversion of CB CD4+ cells [38], our results demonstrated rapid decrease of CD45RA+CD45RO- cells with reciprocal increase in CD45RA-CD45RO+ cells in both the CD4+ and CD8+ subsets when CBMC or purified CD3+ cells were stimulated by PHA with or without IGF-I. As supported by the kinetics of expression, especially the presence of the transitional dual positive cells, the observed decline in naïve cells and corresponding increase in mature cells represent the conversion of CD45 isoforms rather than the preferential expansion/survival of mature cells or the selective elimination of naïve cells.

The PHA-induced *in vitro *CB T cells maturation was delayed in the absence of accessory cells. This delay in maturation might have led to delayed expression of IGF-I receptors, and hence, IGF-I only slightly promoted T cell maturation. Previous studies on the expression of IGF-I receptors on activated T cells were limited to adult cells and the results have been controversial. Using iodopeptide comparative binding studies, an increase in IGF-I receptors expression on activated T cells has been reported [39]. Different IGF-I receptor expression on activated T cells (CD4+CD45RA+ > CD8+CD45RA+ > CD8+CD45RO+ > CD4+CD45RO+) has also been reported [40]. On the contrary, Schillaci et al [41] reported a rapid decline in IGF-I receptors on both CD4+ and CD8+ cells after activation. Further experiments are therefore needed to determine if the effect of IGF-I in promoting the change in CD45RA to CD45RO isoforms in neonatal cells is due to differential expression of IGF-I receptors in these subpopulations.

Due to the limitation of two colour flow cytometry or the combination of antibodies in the past, some reports have considered the expression of CD45RA and CD45RO to be mutually exclusive [13,14]. Only few reports have reported the presence of dual positive CD45RA+CD45RO+ in adult peripheral blood. These cells were proposed to be representing activated lymphocytes after viral infection [42] or primed transitional cells with characteristic antigenic and cytokine expression [43]. In a study on neonates with documented sepsis, up regulation of CD45RA+RO+ cells on CD4+ cells was detected early in the infection [44]. In our study, we have consistently detected a CD45RA+CD45RO+ population in the activated culture. The different kinetics of dual positive cells in CBMC and purified T cell culture suggested that some factors which promote a rapid but transient increase of dual positive cells in Day 3 CBMC was missing in purified T cell culture (Figure 1). This hypothesis was subsequently confirmed by the co-culture experiments, in which the addition of monocytes to T cells at 1:4 ratio restored the kinetics of dual positive T cells (data not shown). Further research on the cellular function of dual positive cells and the molecules regulating T cell maturation is important because these cells may be potential candidates for ex vivo expansion of T cells for autologous immunotherapy. The possibility of interconversion of CD45RA+ and CD45RO+ cells also deserves further investigation which may have implication in the strategy of limiting GvHD in haemopoietic stem cell transplant.

In previous study of T cell proliferation, it has been suggested that complete activation of T cells requires at least two signals: (1) by antigen-specific T cell receptor; and, (2) by antigen presenting cells and/or cytokines produced by antigen presenting cells [45]. Some reports also emphasised on an absolute requirement of accessory cells for T cell proliferation [46-48]. The ability of different subpopulations of adult blood mononuclear cells to serve as accessory cells has been studied previously. Halvorsen *et al *[49] have reported that the removal of monocytes decreased the ability of non T cells to serve as accessory cells for both CD4+ and CD8+ cells stimulated by PHA. The need of monocyte to T cell contact has also been established by separating the cells by a semi-permeable membrane which allows the diffusion of macromolecules [50]. Novel pathway of monocyte dependent regulation of T cells has been reported and the relationship between monocytes and T cells is complicated because the molecules expressed on monocytes can be stimulatory, such as CD1a [51], or inhibitory, such as CD98 [52]. In this study, purified autologous CD14+ monocytes added to purifed CD3+ T cells promoted the PHA-induced T cell maturation and further increased the percentage of CD45RA-CD45RO+ cells in Day 6 culture. The addition of monocytes to PHA-stimulated CD3+ cells in the presence of IGF-I, significantly increased the percentage of CD45RA-CD45RO+ cells. We, therefore, suggest that IGF-I acts on monocytes which in turn promotes the PHA-induced CD45 isoform conversion in CB T cells.

The ratio of monocyte to T cells in the co-culture may also affect the PHA- induced CD45 isoform conversion. In this study, we have mixed the cells at monocyte to T cell ratio of 1:4 based on the ratio observed in freshly isolated CBMC. In other studies, the addition of only 2–4% mitomycin C treated plastic adherent CD14+ monocytes was shown to be effective in inducing activation of T cells [38,53]. On the other hand, Wesch et al [54] have reported significant cell death in a 1:1 co-culture of T cells and monocytes in the presence of PMA or PHA. The CD4+ and CD8+ subsets were equally susceptible to the monocyte dependent cell death (MDCD) but the effect of different mitogens is regulated by different mechanisms. We have not studied the effect of monocytes on T cell death but the observation under light microscopy and the scatter characteristics determined by flow cytometry did not show obvious difference in cultures with and without monocytes.

The relationship between cytokine production and cellular activation in the co-culture system is multidirectional. When stimulated, cord blood cells can produce IL-6, IL-2, IFN-γ, TNF-a and IL-10 but little IL-4 and IL-5 [9,55,56]. Freshly purified CD4+CD45RA+ cells, however, did not produce IL-2, IL-4 and IFN-γ upon primary stimulation (PHA or anti-CD2) but secrete IL-2 and IFN-γ after secondary stimulation by accessory cells [38,55]. IFN-γ can in turn stimulate HLA-DR expression on monocytes further enhancing their accessory function [57]. It is therefore important to study the cytokines released in the co-culture of monocytes and T cells in our system for the identification of potential candidates responsible for the observed induction in T cell maturation.

It has been reported that IGF-I stimulates the release of cytokine from monocytes [27] and it has been shown to stimulate the release of significantly increased amounts of IL-6 in PHA-induced CBMC culture in our previous report [9]. IL-6 is produced by a variety of haemopoietic cells and the cross-linking of Fc receptors on monocytes and triggers IL-6 production [58]. IL-6 provides accessory signals for T cell activation, and antisera to IL-6 completely neutralised the helper effect of monocyte supernatant for PHA-induced human T cell activation and proliferation[59,60]. In this study, IGF-I only stimulated a very low level of IL-6 in the purified CD3+ T cells suggesting that the major source of IL-6 in CBMC culture is from non-T cells. Interestingly, the addition of IL-6 (in the range of 1 – 50 ng/mL) did not alter the kinetics nor the extent of PHA-induced CB T cell maturation. This is suggestive that CB T cell maturation may require the presence of other cytokines and/or cell-cell interaction. Future experiments using transwell to separate the T cells and monocytes in the co-culture can determine if the effect of monocyte is contact dependent.

Activation of T cells by PHA leads to a series of events including maturation, proliferation and activation induced apoptosis. We have previously reported the anti-apoptotic effect of IGF-I and demonstrated the downregulation of Fas expression on PHA-induced T cells in CBMC. In the purified CB CD3+ T cells culture, the anti-apoptotic effect of IGF-1 is weaker than that observed in the CBMC culture. It is evident that factors that are present in CBMC culture but absent in the purified T cell culture are responsible in mediating the anti-apoptotic effect of IGF-1. We showed that IL-6, a factor that is upregulated by IGF-1 in CBMC culture [9], strongly inhibited PHA-induced apoptosis in purified T cell culture (Figure 5). The concentration of IL-6 used (10 ng/mL) is comparable to the maximum concentration assayed in the CBMC culture (~5 ng/mL).

The anti-apoptotic role played by IL-6 was further confirmed by the neutralising experiments. As shown in Figure 6, the neutralisation of IL-6 in the PHA + IGF-I CBMC culture significantly increased the percentage of apoptotic cells. Since the anti-apoptotic effect of IGF-I was not completely neutralised by anti-IL-6 antibodies, the result suggested that other cellular or molecular factors in the mixed CBMC culture may be mediating the effects of IGF-I. The addition of other neutralising antibodies may allow us to identify these factors.

It has been reported that IL-6 prevents the TCR/CD3 activation induced cell death by decreasing the expression of Fas and FasL expression [61]. We, therefore, determined whether the anti-apoptotic effect of IL-6 is mediated through the downregulation of Fas expression in the CD3+ T cell culture. To our surprise, IL-6 slightly upregulated Fas expression (Figure 7) but the induction did not reach statistical significance. This result suggested that (1) the anti-apoptotic effect of IL-6 in purified CD3+ T cell culture is not Fas dependent, and (2) cell-cell interaction and other cytokines upregulated by IGF-1 may be responsible for the down regulation of CD95 (Fas) previously observed in the CBMC culture. In addition, IL-6 has also been reported to protect cells from apoptosis via the bcl-2/p53 pathway [62]. Therefore other intracellular signalling pathway and the expression of IL-6 receptor on CB T cell subsets also worth further investigation.

## Conclusion

In this study, we have described the role of accessory cells and IL-6 in mediating the effect of IGF-I on PHA-induced CB T cells maturation and apoptosis. Knowledge on the mechanism by which monocytes play their accessory role may be used to develop new protocols for therapeutic manipulation of cells. With the high frequency of neonatal sepsis and increased use of CB for haemopoietic reconstitution, a better understanding of CB T cell physiology and the potential of some cytokines in promoting the survival and activation of T cells will help to lower the mortality caused by infection in infants [63-66]. The knowledge will also give insights into designing *ex-vivo *expansion of cells for immunotherapy and graft engineering.

## Abbreviations

IGF-I: insulin-like growth factor I; CB: cord blood; MC: mononuclear cells; PHA: phytohaemagglutinin; IL: interleukin.

## Authors' contributions

HKW Law, W Tu, and YL Lau designed research. HKW Law, W Tu and E Liu performed research. HKW Law, W Tu and YL Lau analyzed data. HKW Law and YL Lau wrote the paper.
